# Eye tracking metrics and leader’s behavioral performance during a post-partum hemorrhage high-fidelity simulated scenario

**DOI:** 10.1186/s41077-021-00156-2

**Published:** 2021-02-04

**Authors:** Emanuele Capogna, Giorgio Capogna, Denise Raccis, Francesco Salvi, Matteo Velardo, Angelica Del Vecchio

**Affiliations:** EESOA Simulation Center, via Giulia di Gallese 15, 00151 Rome, Italy

**Keywords:** High-fidelity scenario, Behavioral skills, Eye tracking

## Abstract

**Background:**

The use of eye tracking in the simulated setting can help improve our understanding of what sources of information clinicians are using as they deliver routine patient care.

The aim of this simulation study was to observe the differences, if any, between the eye tracking patterns of leaders who performed best in a simulated postpartum hemorrhage (PPH) high-fidelity scenario, in comparison with those who performed worst.

**Methods:**

Forty anesthesia trainees from the University of Catania Medical School were divided into eight teams, to enact four times the same scenario of a patient with postpartum hemorrhage following vaginal delivery.

Trainees who were assigned the leader’s role wore the eye tracking glasses during the scenario, and their behavioral skills were evaluated by two observers, who reviewed the video recordings of the scenarios using a standardized checklist. The leader’s eye tracking metrics, extracted from 27 selected areas of interest (AOI), were recorded by a Tobii Pro Glasses 50 Hz wearable wireless eye tracker. Team performance was evaluated using a PPH checklist.

After completion of the study, the leaders were divided into two groups, based on the scores they had received (High-Performance Leader group, HPL, and Low-Performance Leader group, LPL).

**Results:**

In the HPL group, the duration and number of fixations were greater, and the distribution of gaze was uniformly distributed among the various members of the team as compared with the LPL group (with the exception of the participant who performed the role of the obstetrician).

The HPL group also looked both at the patient’s face and established eye contact with their team members more often and for longer (*P* < .05). The team performance (PPH checklist) score was greater in the HPL group (*P* < .001).

The LPL group had more and/or longer fixations of technical areas of interest (*P* < .05).

**Conclusions:**

Our findings suggest that the leaders who perform the best distribute their gaze across all members of their team and establish direct eye contact. They also look longer at the patient’s face and dwell less on areas that are more relevant to technical skills. In addition, the teams led by these best performing leaders fulfilled their clinical task better. The information provided by the eye behaviors of “better-performing physicians” may lay the foundation for the future development of both the assessment process and the educational tools used in simulation.

**Trial Registration:**

Clinical.Trial.Gov ID n. NCT04395963.

## Background

Eye tracking is the process of measuring eye movements, using a device called an eye tracker, to register attention behavior while performing a task.

The principle underlying the use of eye tracking technology is the “eye–mind” theory [1], that suggests there is a relationship between where the individuals look (fix their gaze) and the insight into the cognitive processes that guide this scanning, essentially what they are paying attention to or thinking about at that point in time.

Although cognitive processes are complex, and it is possible that an individual may be simultaneously fixating on one thing but thinking about something else, studies have demonstrated that an individual’s attention and thoughts at a given point in time most likely correspond to their point of fixation [1, 2].

In the medical field, perception and visual expertise have a high impact on work efficiency and effectiveness, as well as on the correctness of analysis and diagnosis [3].

Eye tracking is able to provide reliable quantitative data, which can be interpreted to give an indication of clinical skill, provide training solutions, and aid in feedback and reflection.

Overall eye tracking methodology has contributed significantly to training assessment and has been used in simulation practices in the attempt to better understand insights into how data are collected and acted on during high-load cognitive processes [4–8].

The use of eye tracking in the simulated setting can help improve our understanding of what sources of information clinicians are and are not using as they deliver routine patient care. Using these data, one can then identify what sources of information individuals who are not making errors are using and in what sequence these individuals are gathering that information.

The aim of this simulation study was to observe the differences, if any, between eye tracking metrics of leaders who performed best in a simulated postpartum hemorrhage (PPH) high-fidelity scenario, in comparison with those who performed the worst.

## Method

The authors registered the study at Clinical.Trial.Gov (ID n. NCT04395963).

Forty PGY4-5 anesthesia trainees volunteered from the University of Catania Medical School to be enrolled in this prospective, observational study. Each participant gave informed written consent, and privacy, confidentiality, and anonymity were fully guaranteed by the EESOA Research Board.

In our region, simulation centers do not have access to a formal ethical approval process.

Our simulation center adheres and follows the Healthcare Simulationist Code of Ethics supported by the Society for Simulation in Healthcare [9]. Our study was eligible for exemption, in accordance with US Federal Human Subject Regulations–Protection of Human Subjects, due to the nature of the study itself, as no patients were involved, the trainees participating were volunteers, the researchers ensured that those taking part in the research would not be caused distress. All participants’ personal and other data were completely anonymized, and all the investigators had no conflict of interest and were not involved in any of the participants’ university teaching programs.

We studied eight teams, each containing five participants, and the scenario was repeated four times in order to note any difference in behavioral performance in the participants who were given the role of leader. Our study was a typical “high-fidelity simulation with role exchange” [10]. It is well-known how exchanging professional roles helps professionals understand and “put themselves in the shoes” of their colleagues. This technique has a high didactic value as it trains trainees to better understand the points of view of other healthcare professionals participating in the emergency. For the purpose of the study, we randomly assigned the “leader’s role” to the same subject and rotated the others during the four scenarios (assigning in turn the roles of midwife, obstetrician, nurse, and anesthesia trainee) in such a way that at the end of the rotation, each of them had participated with a different role.

For the leader’s role, we selected those who had the most experience in obstetric anesthesia, based on the time spent in the delivery room during their curricular rotations. Thereafter, we randomly gave them the role of “senior anesthesiologist” in each scenario, making sure that among the different roles assigned, the one of “senior” was the only one they interpreted. In this way, we expected that the participant who was assigned the role of “senior anesthesiologist” (and who in fact had the most experience in obstetric anesthesia) would take the leadership. In the case of shared leadership with someone else, the case was not included in the study.

Every trainee who was assigned the leader’s role wore the eye tracking glasses during the scenario.

For this study, we used a commercially available Tobii Pro Glasses 50 Hz wearable wireless eye tracker. This system can measure eye movements using cameras integrated into the eyeglasses which record the corneal reflection of infrared lighting to track pupil position, mapping the subject’s focus of attention on video recordings of the subject’s field of vision (gaze). In addition to tracking gaze, this system also enables the measurement of various eye metrics including fixation frequency and dwell time, used as a surrogate measure of perceived stimulus importance [11].

All the eye-tracked procedures were recorded immediately after accurate individual calibration, during which the participant, after wearing the glasses unit, focused on the center of the calibration target.

**Fig. 1:**
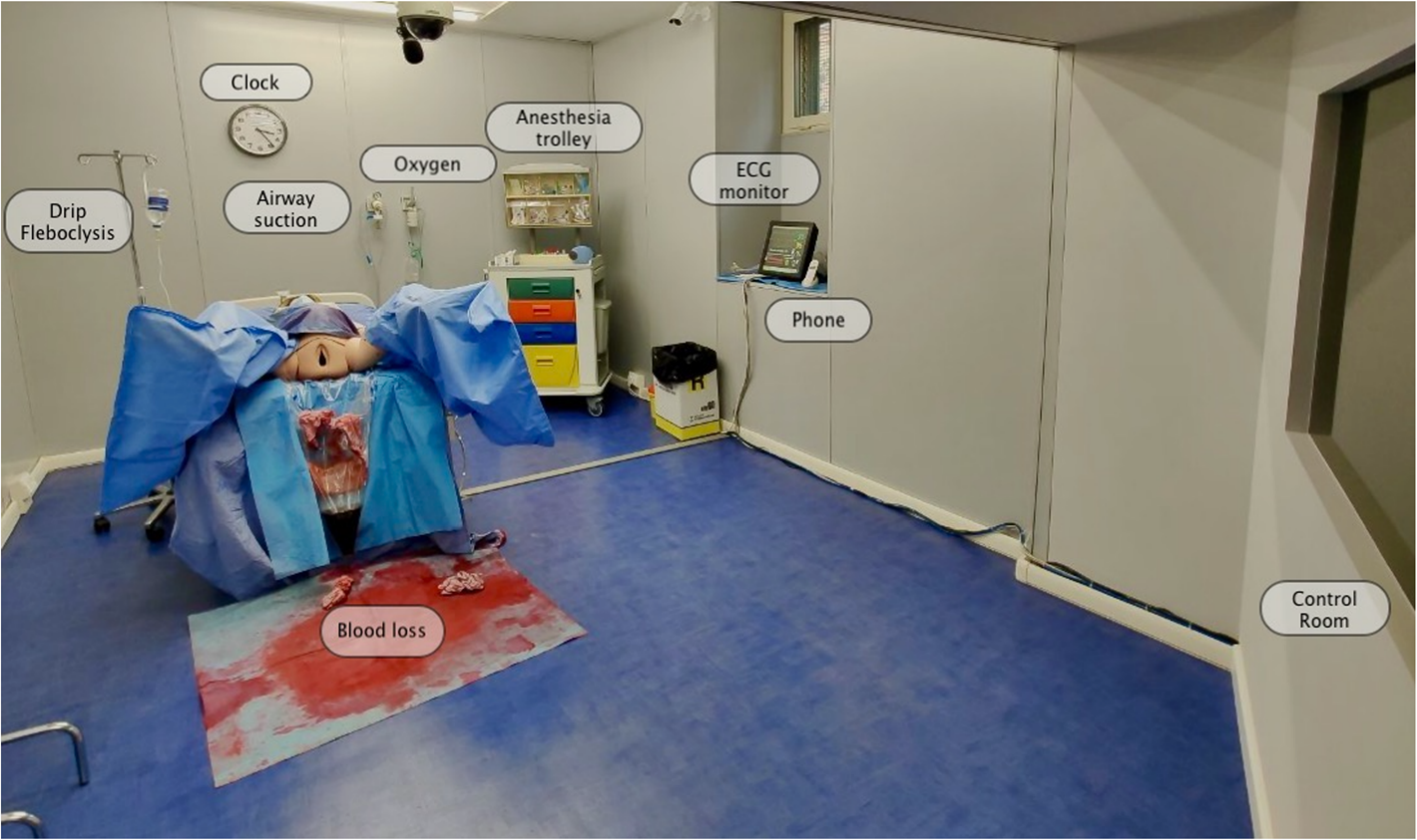
Areas of interest (AOI) concerning the environment/simulation room

**Fig. 2:**
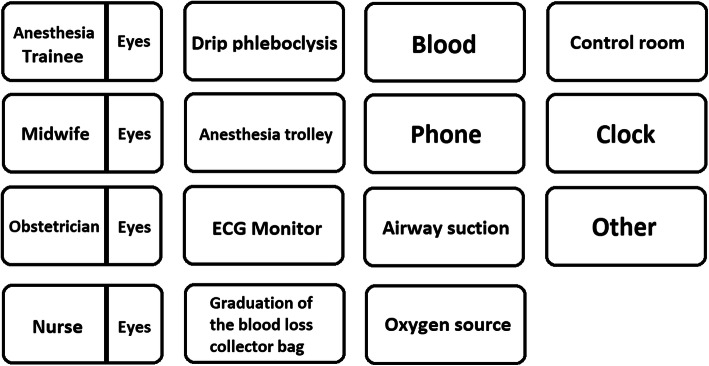
Areas of interest (AOI) concerning the participants and the environment/simulation room

All the eye tracking video recordings were stored and analyzed using the Tobii Pro Lab Software. We selected 27 areas of interest (AOI) (Figs. 1 and 2), to define regions of a displayed stimulus and to extract metrics specifically for those regions as follows:

Eleven AOI concerning the simulation room: airway suction, anesthesia trolley, blood loss, drip phleboclysis, ECG monitor, clock, oxygen source, control room, phone, blood loss collector bag, and other (the space in general) (Figs. 1 and 2)

Eight AOI concerning the participants: anesthesia trainee, anesthesia trainee’s eyes, obstetrician,

obstetrician’s eyes, nurse, nurse’s eyes, midwife, midwife’s eyes (Fig. 2)

**Fig. 3:**
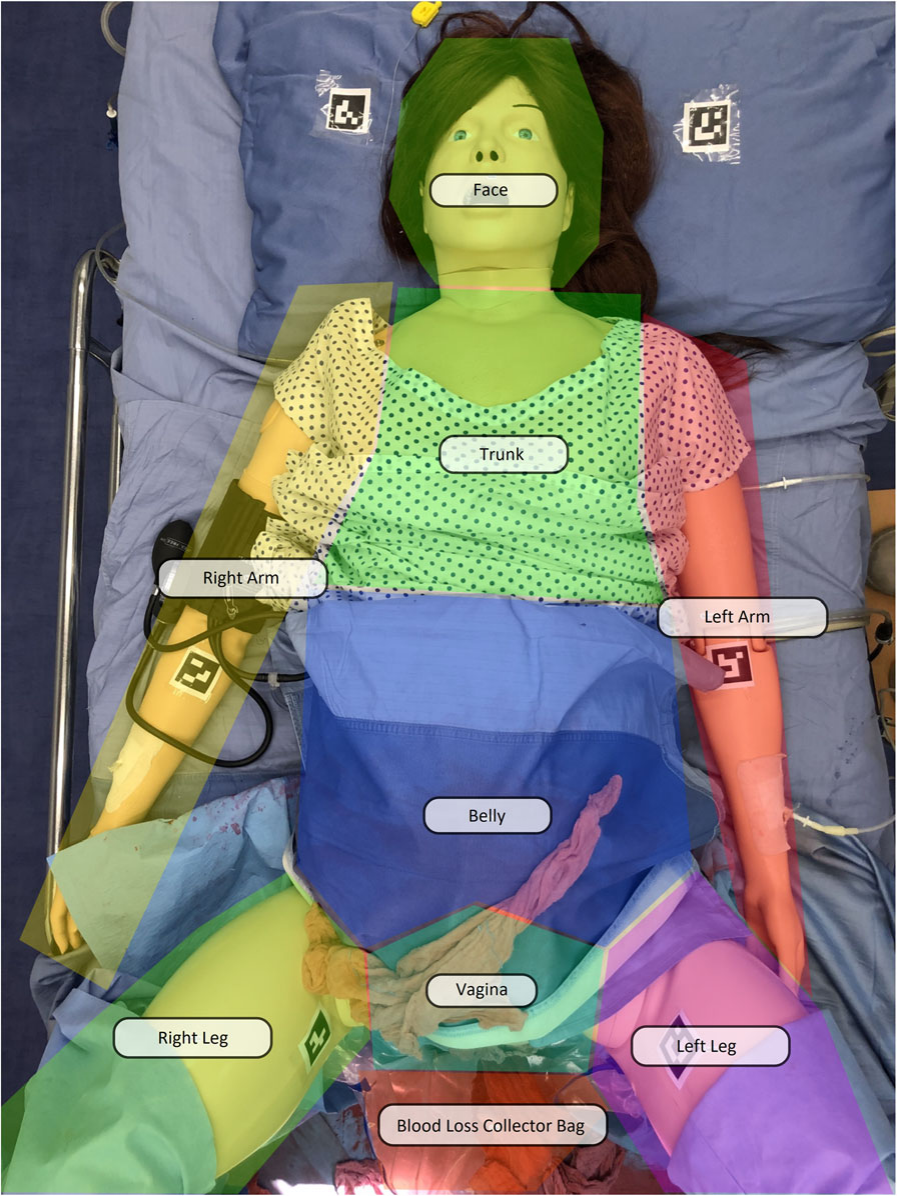
Areas of interest (AOI) concerning the manikin

Eight AOI concerning the manikin: right arm (on which the sphygmomanometer was placed), the left arm (on which two intravenous accesses were set), right leg, left leg, belly, trunk, vagina, face (Fig. 3).

The number and duration of fixations for each area of interest were examined.

The fixation points were generated by the Tobii software’s filter according with the following parameters: max gap length 75 ms; noise reduction: window size (samples) 3; velocity calculator: window length 20 ms; merge adjacent fixations: max time between fixations 75 ms; max angle between fixations 0.5°; minimum fixation duration 60 ms.

Each fixation point was assigned manually to a specific AOI by an independent, blinded investigator (simulation technician specifically trained in eye tracking) who reviewed the video recording of each scenario.

The eye tracking metrics were mapped as gaze plots and heat maps. Heat maps and gaze plots are data visualizations that can communicate important aspects of visual behavior clearly and with great power. Gaze plots show the location, order, and time spent looking at locations on the stimulus. Time spent looking, most commonly expressed as fixation duration, is shown by the diameter of the fixation circles. The longer the look, the larger the circle.

Heat maps show how looking is distributed over the stimulus and can effectively reveal the focus of visual attention.

The evaluation of the behavioral skills of the leader and of the technical skills of the team was made by two expert observers not involved in the scenarios, who independently reviewed the video recordings of the scenarios.

The technical skills of the team were evaluated on the basis of the completion of a PPH checklist. For the design of this checklist, we reviewed PPH guidelines from recognized obstetric bodies and literature, relevant papers from the literature, and their institutional PPH protocol [12–15].

We then chose, by consensus, the final action items for the checklist, identifying 25 standardized key tasks for inclusion on the PPH checklist. We assigned one point for each task executed, for a maximum of 25 points. This checklist worked as the reference guide for pre-scenario briefing and for the team’s technical skills evaluation during the scenario (Appendix 1).

We also developed a standardized questionnaire for the evaluation of the behavioral skills of the leader (Appendix 2), derived from the Anaesthetists’ Non-Technical Skills (ANTS) behavioral marker system [16] and the Ottawa Global Rating Scale (GRS) [17]. Each independent observer assigned a score for leadership, communication, and situational awareness (Appendix 2).

Interobserver reliability was also calculated

No formal training took place before the first scenario, in order to consider the first scenario as the participants’ baseline performance. All the teams underwent standardized educational training on PPH Guidelines immediately before the second, third, and fourth scenarios.

The scenario consisted of a severe PPH (> 1500 mL blood loss) due to refractory uterine atony in a multiparous 28-year-old patient who had undergone a spontaneous vaginal delivery. The patient became tachycardic and hypotensive consistent with hemorrhagic shock. All simulations were performed in the simulation room of the EESOA Simulation Center (Rome) using a high-fidelity manikin (Sim Mom Maternal and Neonatal Birthing Simulator, Laerdal, Norway). All scenarios were videotaped. The scenario was stopped when each team had completed all 25 tasks of the checklist, or when 15 min had elapsed. Each scenario was followed by a standardized debriefing led by an expert debriefer.

A study investigator, expert in both PPH and simulation debriefing and not involved in the simulation activity, observed each scenario in the control room, to record and check the team’s performance (PPH evaluation and treatment), according to the established 25 PPH key tasks (Appendix 1). The leaders’ behavioral scores (Appendix 2) were assigned by two observers, experts in communication and evaluation in simulation and not involved in the scenarios, who reviewed the videos of each simulation.

After completion of the study, all the leaders were divided into two groups, depending on the scores received for their leadership behavioral skills during the scenarios, and their eye tracking metrics were compared. We divided all the leadership situations into two groups: the High-Performance Leader group, HPL, which included all the leaders who had received the highest scores (score = 5) and the Low-Performance Leader group, LPL, which included the leaders who had received the lowest scores (score: 1–2) during the four scenarios.

### Statistical analysis

Data are presented as means, confidence intervals (95% CI), and standard deviations (SD).

The leaders’ performances were calculated by using the means of the scores given by each independent observer on leadership, communication, and situational awareness (Appendix 2).

In order to better discriminate the best and worst performance assessment, a linear transformation to convert the scale into a 5-point scale was used.

The eye tracking metrics were compared by using a two-way unpaired *t* test with lower and higher alternative hypothesis to compare the two groups.

The overall team performance (assessed by the PPH checklist) from the first to the fourth scenarios was examined by using the ANOVA test and Dunnett’s post hoc test.

It was not possible to calculate the sample size *a priori* because at the start of the study, it was obviously unreasonable to determine how many leaders would perform well or poorly.

The post hoc power analysis, set at a significance level of 0.95 and a calculated effect size for almost all comparison above 1, was in the range of 60–80% power.

The Cohen’s Kappa coefficient was applied to measure the degree of agreement between the two assessors (inter-rater reliability).

## Results

All the participants successfully completed the scenarios. Out of a total of 32 planned “leadership situations” (performed by each leader of the eight groups, playing 4 scenarios), in eight situations, the leaders received the best scores (score = 5) (HPL group) and in seven, the leaders received the lowest scores (1–2) (LPL group). All the other episodes that had an intermediate score, including the cases of shared leadership, or in the case of the leadership being taken by another participant, were not considered for the data analysis. There was a high level of concordance (*k* = 0.92) between the two observers who independently made the evaluations.

**Fig. 4:**
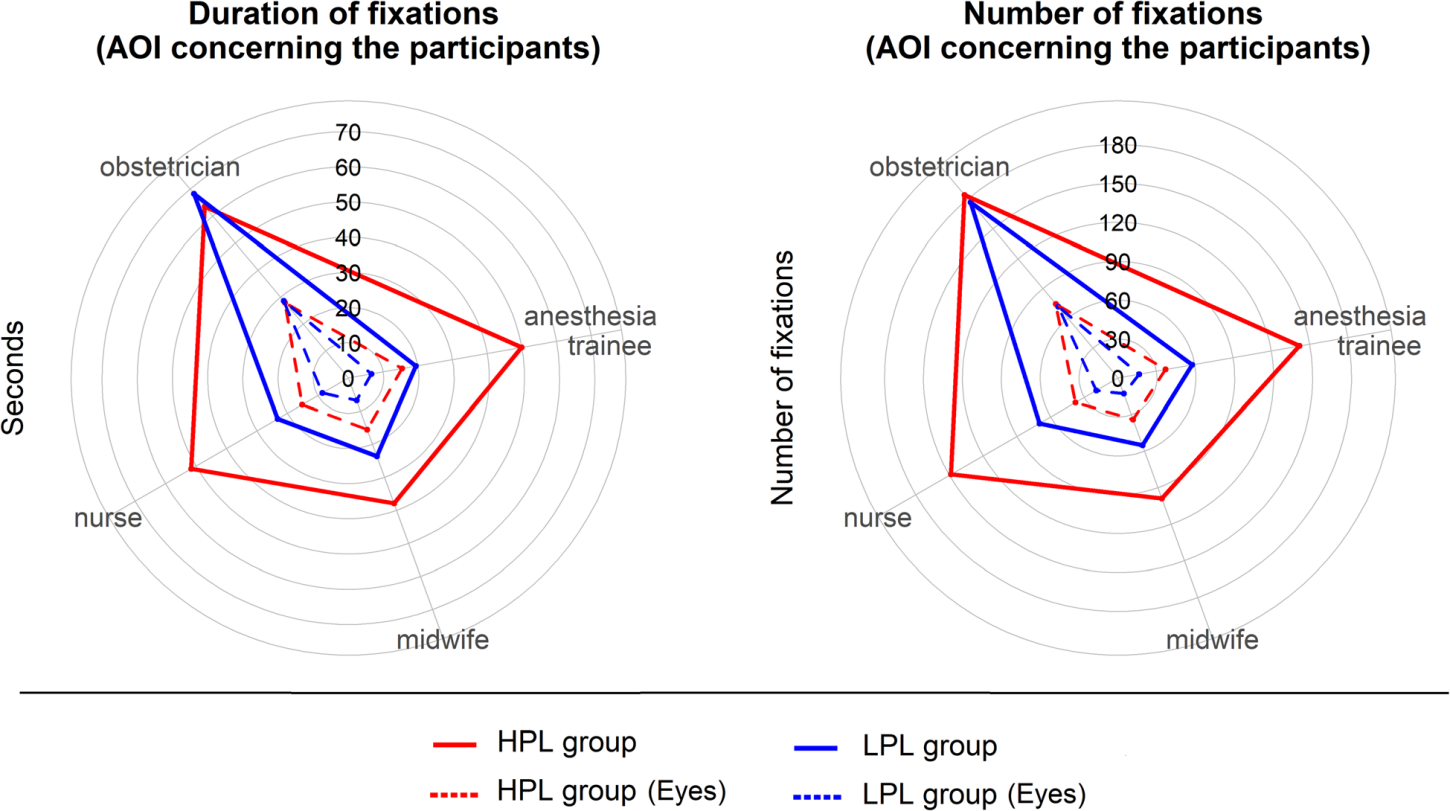
Radar plot describing duration and number of fixations of the leaders on each of the participants in the scenario. *HPL* High-performance leader group, *LPL* Low-performance leader group

In Table 1 and Fig. 4, the duration and number of fixations of the leaders on each of the participants in the scenario are reported.

**Table 1:** Number and duration of fixations of the leader on each of the participants

AOI	LPL	CI 95%	SD	HPL	CI 95%	SD	*t* test
**Number of fixations (count, mean values)**							
Anesthesia trainee	58	(32–84)	28.3	142	(95–189)	69.6	*P* < .001
Anesthesia trainee (eye)	17	(7–26)	10.2	37	(25–49)	17.8	*P* < .001
Obstetrician	177	(97–257)	86.6	184	(153–215)	46.5	*P* > .05
Obstetrician (eye)	72	(29–115)	46.7	74	(59–90)	23.7	*P* > .05
Nurse	70	(40–99)	32.0	149	(123–175)	38.7	*P* < .001
Nurse (eye)	19	(8–31)	12.3	38	(24–51)	20.1	*P* < .01
Midwife	13	(1–25)	12.6	34	(19–49)	23.0	*P* < .01
Midwife (eye)	55	(26–84)	31.5	99	(68–130)	46.4	*P* < .01
**Duration of fixations (seconds, mean values)**							
Anesthesia trainee	26.1	(9–22)	17.7	65.4	(41–89)	35.4	*P* < .001
Anesthesia trainee (eye)	6.7	(1–12)	5.9	15.4	(9–21)	9.5	*P* < .05
Obstetrician	96.8	(61–132)	38.6	91.5	(74–108)	25.8	*P* > .05
Obstetrician (eye)	28.6	(15–41)	13.8	28.0	(21–34)	9.8	*P* > .05
Nurse	31.8	(20–43)	12.3	66.8	(48–85)	27.1	*P* < .001
Nurse (eye)	8.5	(5–12)	4.0	15.6	(9–21)	8.9	*P* < .05
Midwife	6.7	(0–14)	8.4	15.6	(8–23)	10.6	*P* < .05
Midwife (eye)	30.4	(13–47)	18.5	53.5	(34–73)	29.3	*P* < .05

*LPL* Low-Performance Leader Group, *HPL* High-Performance Leader Group

In the HPL group, the average duration and number of fixations were greater, and the distribution of gaze was uniformly distributed among the various members of the team as compared with the LPL group (with the exception of the participant who performed the role of the obstetrician).

The leaders who performed better established eye contact with their team members more often and for longer (with the exception of the participant who performed the role of the obstetrician) than the leaders who performed worse.

**Fig. 5:**
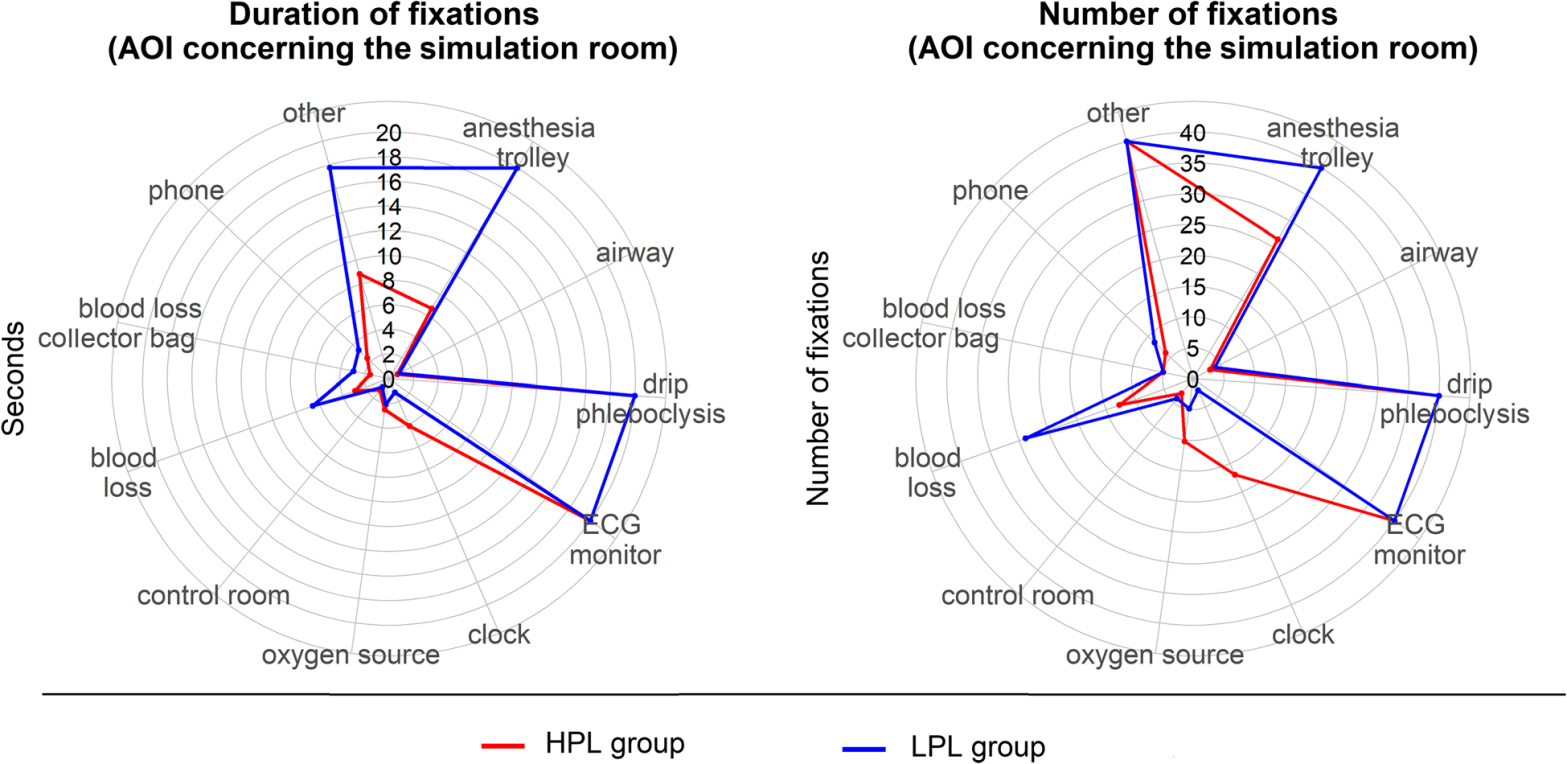
Radar plot describing number and duration of fixation of the leader on the environment, simulation room, and manikin. *HPL* High-performance leader group, *LPL* Low-performance leader group

**Fig. 6:**
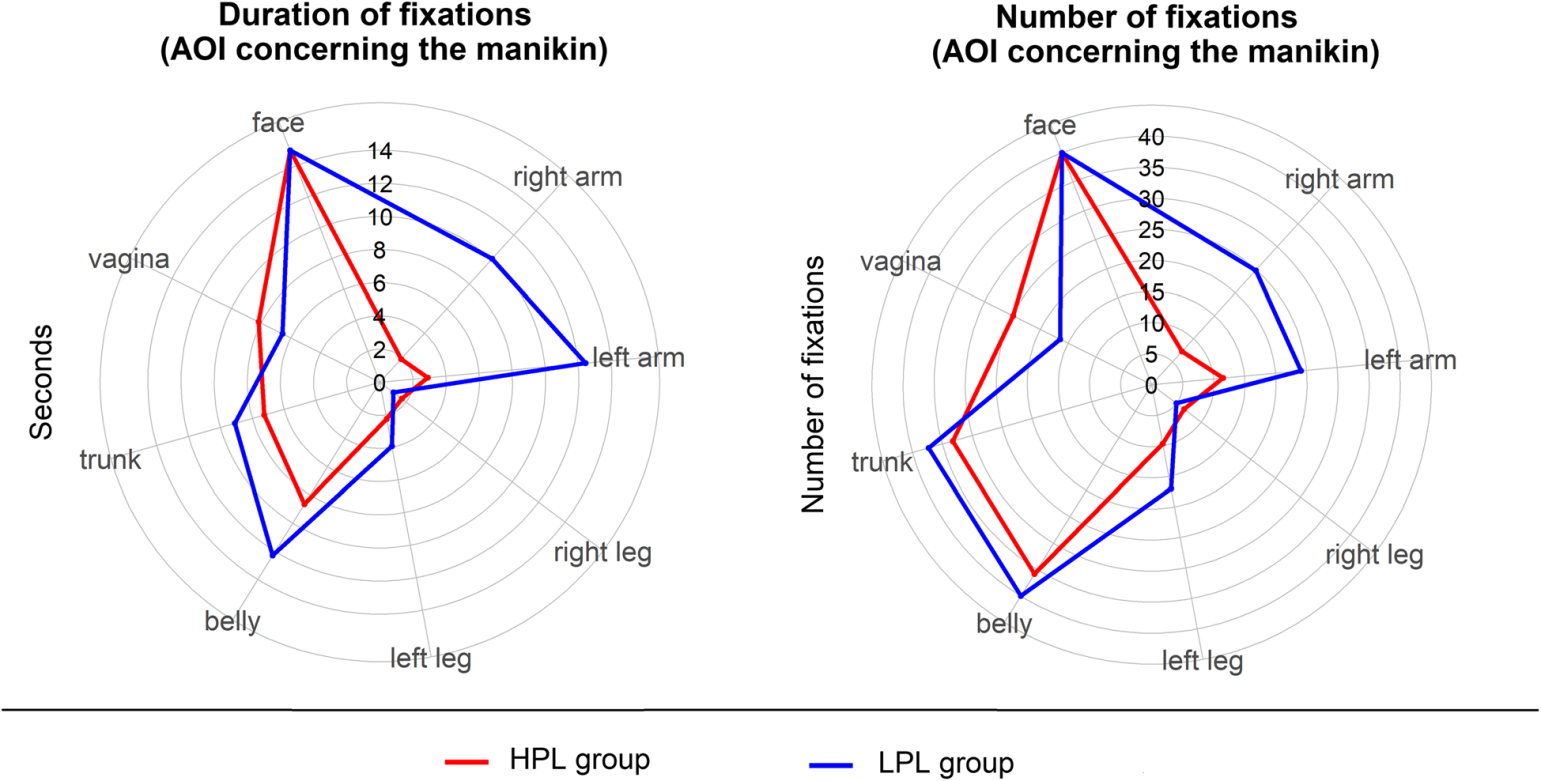
Radar plot describing number and duration of fixation of the leader on the manikin. *HPL* High-Performance Leader Group, *LPL* Low-Performance Leader Group

**Fig. 7:**
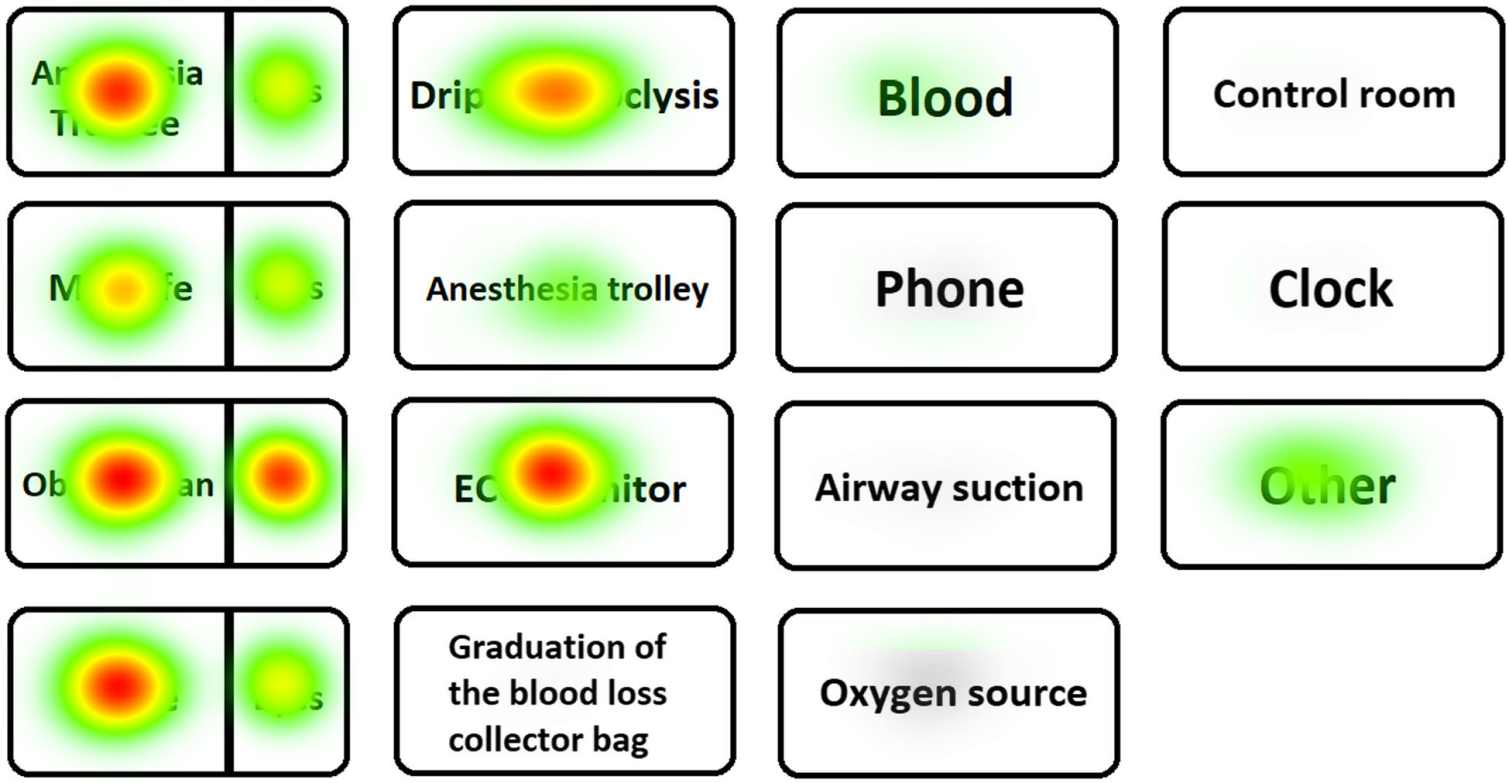
Heat maps of the high-performing leaders. The hotter the point (from green to red), the greater the cumulative fixation time in the region of interest

**Fig. 8:**
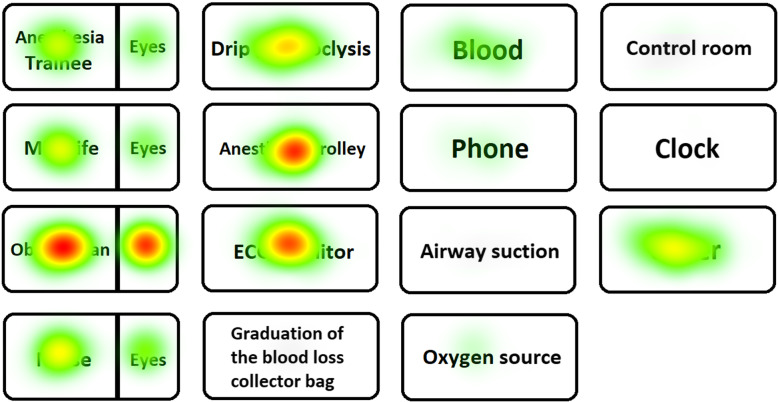
Heat maps of the low-performing leaders. The hotter the point (from green to red), the greater the cumulative fixation time in the region of interest

In Table 2 and Figs. 5 and 6, the number and duration of fixations of the leader on the simulation room and manikin are reported. In Figs. 7 and 8, respectively, the heat maps of the highly performing leaders and of the low-performing ones are reported.

**Table 2:** Number and duration of fixations of the leader on simulation room and manikin

AOI	LPL	CI 95%	SD	HPL	CI 95%	SD	*t* test
**Number of fixations (count, mean values)**							
Right arm	25	(7–43)	19.7	7	(3–12)	5.7	*P* < .05
Left arm	24	(15–33)	9.4	12	(8–16)	5.3	*P* < .05
Right leg	5	(3–7)	2.6	7	(2–11)	6.5	*P* > .05
Left leg	17	(7–27)	10.8	10	(4–16)	8.6	*P* > .05
Belly	44	(19–69)	27.0	36	(23–49)	19.7	*P* > .05
Trunk	37	(24–51)	14.3	33	(27–40)	9.6	*P* > .05
Vagina	16	(10–23)	6.9	25	(12–37)	18.5	*P* > .05
Face	113	(80–145)	35.6	153	(120–195)	62.7	*P* < .05
Airway	4	(0–9)	2.0	3	(0–6)	4.0	*P* > .05
Anesthesia trolley	93	(42–143)	54.4	26	(18–34)	12.0	*P* < .05
Drip phleboclysis	88	(52–124)	38.8	116	(75–156)	60.7	*P* > .05
ECG monitor	99	(82–115)	17.8	114	(90–139)	36.3	*P* > .05
Clock	2	(0–4)	1.6	17	(9–24)	3.1	*P* > .05
Oxygen source	5	(2–8)	3.2	10	(5–15)	7.1	*P* < .05
Blood loss	29	(3–55)	27,8	13	(8–17)	6.9	*P* > .05
Blood loss collector bag	5	(1–9)	4.2	5	(0–11)	3.9	*P* > .05
Other	81	(35–127)	49.7	41	(18–64)	34.4	*P* < .05
Control room	4	(1–7)	3.3	3	(1–5)	1.6	*P* > .05
Phone	9	(4–13)	5.3	6	(2–10)	4.8	*P* > .05
**Duration of fixations (seconds, mean values)**							
Right arm	10.1	(0.9–19.2)	9.9	1.9	(0.3–3.4)	1.9	*P* < .05
Left arm	12.5	(4.2–21.7)	8.9	2.9	(1.6–4.1)	1.6	*P* < .05
Right leg	1.0	(0.6–1.4)	0.4	1.7	(0.3–3.0)	2.0	*P* > .05
Left leg	3.9	(1.6–6.2)	2.5	2.3	(0.6–3.8)	2.2	*P* > .05
Belly	12.3	(4.3–20.2)	8.6	8.6	(5.3–12.0)	4.9	*P* > .05
Trunk	9.1	(4.1–14.0)	5.3	7.3	(5.3–9.21)	2.9	*P* > .05
Vagina	6.6	(2.0–11.2)	4.9	8.2	(4.4–11.9)	5.5	*P* > .05
Face	35.8	(29.6–41.8)	6.6	43.3	(31.6–55.0)	17.4	*P* > .05
Airway	0.9	(0.0–2.7)	0.7	0.8	(0.0–1.5)	1.0	*P* > .05
Anesthesia trolley	31.8	(14.9–48.7)	18.2	6.6	(4.3–9.0)	3.5	*P* > .05
Drip phleboclysis	33.8	(18.2–49.2)	16.8	37.5	(22.5–52.4)	22.3	*P* > .05
ECG monitor	57.6	(45.4–69.7)	13.1	45.7	(33.0–58.3)	18.8	*P* < .05
Clock	1.2	(0.1–1.6)	0.3	4.2	(2.4–5.9)	1.2	*P* > .05
Oxygen source	2.1	(0.1–4.0)	2.1	2.5	(1.4–3.6)	1.6	*P* > .05
Blood loss	6.6	(1.0–12.1)	6.0	2.9	(1.7–4.1)	1.8	*P* > .05
Blood loss collector bag	3.0	(0.0–7.3)	2.7	1.6	(0.3–2.8)	1.4	*P* > .05
Other	17.8	(7.8–27.6)	10.7	8.8	(2.5–15.1)	9.3	*P* < .05
Control room	0.9	(0.2–1.5)	0.7	1.2	(0.0–2.7)	1.2	*P* > .05
Phone	3.4	(1.4–5.3)	2.2	2.4	(0.7–4.1)	2.2	*P* > .05

*LPL* Low-performance leader group, *HPL* High-performance leader group

The leaders who performed worse had more and/or longer fixations on some technical areas of interest, such as the right arm (on which the sphygmomanometer was placed) (*P* < .05), the left arm (on which two intravenous accesses were set) (*P* < .05), and the space in general (*P* < .05). They also fixed their gaze on the anesthesia cart for a longer time (*P* < .05). The number of fixations of the patient’s face was greater in the leaders who performed better (*P* < .05).

The overall team performance (evaluated by the PPH checklist scores) improved from the first to the fourth scenarios, and the mean score (standard deviation) for each scenario was respectively 15.63 (3.42), 18.88 (2.3), 20.2 (2.88), and 21.63 (2.07) (*P* < .05).

The overall team performance score was greater in the HPL group (*P* < .001).

Interestingly, eye tracking video recordings allowed us to notice that no leader looked at the glass window of the control room, at the microphones or at the video cameras of the simulation room, and this may support the full and life-like involvement of the participants in the scenarios.

## Discussion

Many eye tracking studies have been undertaken in simulated clinical settings. However, eye tracking has mostly been used to assess the performance of clinical skills [4, 18, 19] or to compare novice and expert clinician’s visual scan paths when performing a practical task [19, 20]. Eye tracking has also been reported as being an aid during the post scenario debriefing [21, 22].

In one study [23], the performance and human factors in high-fidelity simulation of postpartum hemorrhage were analyzed by the visual behavior analysis using a standardized fixed eye tracking protocol, during viewing of a simulated video. In this study, the authors concluded suggesting a “neuroscientific approach with new technology like eye tracking to provide a new objective and more sensitive insights on human factors in simulated medical emergency situations”. In line with this previous study, we made an attempt to use a mobile (glasses) eye tracking technology to investigate behavioral rather than technical or practical skills during a high-fidelity scenario.

Our findings suggest that there is an association between the leaders’ distribution of gaze across all members of their respective teams, establishing and maintaining eye contact, and their best performance. A prolonged look into the patient’s face and a decreased dwelling on areas that are more relevant to technical skills were also associated with the best performing leaders’ ocular behavior.

The use of eye tracking technology to capture the eye movements of better-performing clinicians during a high-fidelity simulated scenario may help to provide new insight into behaviors associated with early identification of medical errors and adverse events. Knowledge of eye movements may also potentially supplement other methods of collecting insight into best performances, such as direct observation, verbal reports, and thinking out loud. Eye contact with the patient and team members may be a marker of better communication by the team leader, since it is well-known that eye contact (gazing) is a marker and a tool of good communication, and it is perhaps the most powerful way we communicate [24]. Our preliminary results by using the eye tracking technology support the hypothesis that team leaders should establish direct eye contact with their team, in order to better communicate with them. Interestingly, the leaders who performed best also looked at the face of the patient more.

It is more important for the leader to take the human behavior into account, dedicating more verbal and nonverbal attention to the team and the patient, rather than getting lost in technical details. This principle is obvious enough for those who practice and teach simulation, but we believe that our study demonstrates that it is possible, by using the eye tracking technique, to quantify and measure this behavioral attention. Notably, the previously described visual behavior of the leaders was associated with their team’s better technical skill score.

Our study has some limitations. We recognize that as the leader becomes familiar with the team members, the eye contact with them may have changed, and this has to be taken into consideration. However, we still believe that familiarity with the team in the actual life may be a value to highlight and not necessarily a limitation.

We recognize that the experimental situation created for our study is not exactly the one that occurs in real life, when an obstetric emergency usually requires a shared leadership between senior anesthesiologist and senior obstetrician, and that therefore our results cannot be fully extrapolated to clinical practice. The purpose of our study, and consequently its design, was to observe changes in the eye tracking metrics of the leader and not the relationship between the leaders in a shared leadership situation, which requires a much more complex design than ours. Nevertheless, we believe our results may have a value in supporting the eye tracking method as a possible additional tool for the observation of the participants in a simulated scenario.

We also recognize that as much as we have tried to homogenize the clinical evolution of the scenario, a definite standardization was not possible due to the nature of the life-like high-fidelity methodology used.

We are aware that both eye tracking and human behavior analysis are subject to a number of variables that are very difficult to control in a simulation environment. We, however, believe that they could nevertheless present an opportunity to quantify some of these communication variables.

## Conclusions

In conclusion, our findings suggest that the leaders who perform the best distribute their gaze uniformly across all members of their team and establish direct eye contact. They also look longer at the patient’s face and dwell less on areas that are more relevant to technical skills. In addition, the teams led by these best-performing leaders fulfilled their clinical task better. This is the first study that has used mobile eye tracking technology to investigate behavioral rather than technical or practical skills during a high-fidelity scenario. The information provided by the eye behaviors of “better-performing physicians” may lay the foundation for the future development of both the assessment process and the educational tools used in simulation.

## Data Availability

The datasets used and/or analyzed during the current study are available from the corresponding author on reasonable request**.**

## Appendix 1: Atonic PPH checklist (Team’s performance)

- □ Call for help
- □ ABCD
- □ Vital signs assessment
- □ Inform patient & relatives
- □ IV – place 2nd large bore
- □ 4T assessment (evaluation of causes of PPH: tone, tissue, trauma, thrombin)
- □ Blood loss evaluation
- □ Stat Labs: CBC + coagulation + TEG
- □ Fundal massage
- □ Bimanual compression
- □ Place Foley
- □ Bakri balloon
- □ Oxytocin
- □ I Line uterotonics
- □ Oxygen
- □ Fluids
- □ Vasopressors
- □ Tranexamic acid
- □ Warming
- □ II Line uterotonics
- □ Blood gas analysis
- □ Transfusions: blood, fresh frozen plasma, cryoprecipitates
- □ Fibrinogen
- □ Consider OR
- □ Consider Intensive Care Unit/ Interventional Radiology

## Appendix 2: Leader’s assessment

### Leadership

Did the Leader Anesthesiologist coordinate everyone’s work?

4 = Always; 3 = Often; 2 = Sometimes; 1 = Never

Did the Leader Anesthesiologist set priorities?

4 = Always; 3 = Often; 2 = Sometimes; 1 = Never

Did the Leader Anesthesiologist distribute the workloads to the team members?

4 = Always; 3 = Often; 2 = Sometimes; 1 = Never

### Situational awareness

Did the Leader Anesthesiologist anticipate the clinical problems?

4 = Always; 3 = Often; 2 = Sometimes; 1 = Never

Did the Leader Anesthesiologist listen to all the suggestions given by the members of the group?

4 = Always; 3 = Often; 2 = Sometimes; 1 = Never

Did the Leader Anesthesiologist reassess the patient’s condition several times?

4 = Always; 3 = Often; 2 = Sometimes; 1 = Never

### Communication

Did the Leader Anesthesiologist share his intervention strategy, in terms of diagnosis and therapy, with the rest of the team?

4 = Always; 3 = Often; 2 = Sometimes; 1 = Never

Did the Leader Anesthesiologist communicate succinctly and clearly?

4 = Always; 3 = Often; 2 = Sometimes; 1 = Never

Did the Leader Anesthesiologist look you in the eye when you spoke to him and/or did you call him by his name?

4 = Always; 3 = Often; 2 = Sometimes; 1 = Never

## Abbreviations

PPH: Postpartum hemorrhage
AOI: Areas of interest
ANTS: Anaesthetists’ Non-Technical Skills
GRS: Ottawa Global Rating Scale
